# UVB suppresses PTEN expression by upregulating *miR-141* in HaCaT cells^☆^

**DOI:** 10.1016/S1674-8301(11)60017-1

**Published:** 2011-03

**Authors:** Wei Li, Wu Di, Lijuan Hua, Bingrong Zhou, Ze Guo, Dan Luo

**Affiliations:** Department of Dermatology, the First Affiliated Hospital of Nanjing Medical University, Nanjing, Jiansu 210029, China

**Keywords:** MicroRNA-141, PTEN, UVB, apoptosis, HaCaT cells

## Abstract

MicroRNAs (miRNAs) are 21 to 24 nucleotide, non-coding RNA molecules that post-transcriptionally regulate the expression of target genes. Ultraviolet B (UVB) radiation has been shown to inhibit phosphatase and tensin homolog deleted on chromosome 10 (PTEN) expression in HaCaT cells through an unknown mechanism. In this study, we investigated whether miR-141 can regulate UVB exposure-mediated inhibition of PTEN expression. Real-time RT-PCR, annexin V/fluorescein isothiocyanate staining, Western blotting and anti-miRNA oligonucleotide transfection were employed in this study. We found that upregulation of *miR*-*141* expression after UVB irradiation was inversely correlated with PTEN expression levels in HaCaT cells. Furthermore, *miR*-*141* expression increased apoptosis, while anti-miR-141 partly restored PTEN expression and reversed the pro-apoptosis effect of UVB. UVB suppresses the expression of PTEN by upregulating *miR*-*141* in HaCaT cells. Therefore, *miR*-*141* is a potential gene therapy target for UVB-induced photodamage.

## Introduction

Ultraviolet (UV) radiation from the sun, particularly the UVB component (290-320 nm), is the major cause of skin cancer. In addition, UV radiation elicits a variety of adverse effects, such as erythema, sunburn, inflammation, hyperplasia, hyper-pigmentation, immunosuppression, premature skin aging, and photocarcinogenesis[1],[2]. These cellular events are mediated by the activation or suppression of a variety of genes after UVB exposure[3]. However, the precise molecular events involved in UVB-regulated gene expression remain largely unknown.

MicroRNAs (miRNAs) are short, noncoding RNAs of approximately 22 nucleotides, which regulate gene expression through sequence-specific base pairing with the 3′-untranslated region (3′-UTR) of target mRNAs[4],[5]. To elucidate the molecular mechanisms underlying photodamage and skin carcinogenesis by UVB, several groups have investigated the microRNA expression profiles of UVB-irradiated cells using microRNA microarray[6]–[8]. For instance, Guo *et al*.[6] investigated the differential expression profiles of miRNAs in NIH 3T3 cells in response to UVB irradiation. Furthermore, changes in miRNA expression and stress granule formation were found to be most pronounced within the first hours after UVB irradiation, suggesting that miRNA-mediated gene regulation operates earlier than most transcriptional responses[7]. The function of the miRNA response may have some relationship with DNA damage response and cell proliferation[7]–[9]. In our preliminary study, we assessed the effect of UVB irradiation on miRNA expression changes in HaCaT keratinocytes and found that 3 miRNAs (miR-188-5p, miR-223 and miR-22) were down-regulated, while 3 miRNAs (miR-125a-5p, miR-146a and miR-141) were up-regulated in UVB-irradiated cells compared with untreated cells. These results indicated that miRNAs were potentially involved in the pathogenesis of UVB-induced photodamage.

MiR-141 is a member of the miR-200 family and is an important regulator of the epithelial to mesenchymal transition[10]–[12]. In addition to the role of miR-141 in the phenotypic conversion of normal cells, dysregulation of miR-141 expression occurs in multiple types of cancer cells and is linked to tumor progression[13]–[15]. However, the role of miR-141 expression in UVB-treated cells is not fully understood. The tumor suppressor phosphatase and tensin homolog deleted on chromosome 10 (PTEN) has been shown to be a target of miR-141 in nasopharyngeal carcinoma cells[15]. Furthermore, both UVA and UVB have been shown to downregulate PTEN in primary human keratinocytes, human HaCaT keratinocytes, human dermal fibroblasts and mouse skin[16],[17]. Decreased PTEN expression after UV irradiation may promote the survival of epidermal keratinocytes[17]. Previous work suggests that PTEN downregulation can occur via two mechanisms: 1) PTEN is cleaved by active caspase in apoptotic cells; 2) PTEN transcription is suppressed in surviving cells independent of caspase activation[17]. In this study, we find that UVB suppresses the expression of PTEN by upregulating miR-141 in HaCaT cells.

## MATERIALS AND METHODS

### Cell culture and UVB irradiation

The immortalized keratinocyte HaCaT cell line was obtained from Dr. Gu, Department of Dermatology, Changhai Hospital, Shanghai, China. Cells were maintained in Roswell Park Memorial Institute (RPMI)-1640 medium with 10% fetal bovine serum and 100 units/mL penicillin/streptomycin at 37°C in 5% CO^2^. At 70%-80% confluence, cells were cultured in serum-free RMPI-1640 medium 24 h before UVB irradiation. Subconfluent cultures were sham or UV-irradiated in phosphate-buffered saline (PBS) through the plastic dish covered with a solar simulator (Sigma, USA). The 1 kW xenon arc lamp was adjusted to 8×10^−5^ W/cm^2^ and metered with a research radiometer fitted with a UVB probe at 285±5 nm. Sham cultures were handled identically except that they were shielded with aluminum foil during the irradiation.

### miRNA isolation and real**-**time RT**-**PCR

Total RNA was extracted from each sample using a TRI Reagent Kit (Ambion, TX, USA) according to the manufacturer's instructions. Expression of miR-141 after UVB irradiation at different dosages (30, 60 and 90 mJ/cm^2^) was analyzed using the TaqMan MicroRNA Assays, TaqMan MicroRNA RT Kit and Taqman 29 Universal PCR Master Mix without UNG Amperase (Applied Biosystems, CA, USA) on an Applied Biosystems 7500 Fast Real Time PCR System. The reactions were incubated in a 96-well plate at 95°C for 10 min followed by 40 cycles of 95°C for 15 s and 60°C for 1 min. The Applied Biosystems 7500 Fast software was used to analyze the Ct values of different miRNAs normalized to an endogenous control (U6). The normalized values (dCT) from Group 1 were then compared to Group 2, yielding miRNA differential expression profiles. The relative amount of miRNAs was determined with the 2^−ΔΔCt^ method.

### Annexin V/fluorescein isothiocyanate staining

Apoptosis levels were detected 24 h after UVB irradiation in all groups. Treated cells were isolated, washed, and stained using annexin V-fluorescein isothiocyanate (FITC) and propidium iodide (PI). Apoptosis was measured by performing flow-cytometric analysis using an ELITE ESP flow cytometer (Beckman-Coulter, USA).

### Western blotting assay

PTEN, phospho-AKT and Bcl-xL protein levels were detected 24 h after UVB irradiation in all groups. Cellular lysate was prepared using radioimmunoprecipitation assay (RIPA) buffer with protease inhibitors and quantified using the bicinchoninic acid (BCA) protein assay (Pierce, IL, USA). Equal amounts of protein were separated by 10% sodium dodecyl sulfate-polyacrylamide gel electrophoresis (SDS-PAGE) and electrophoretically transferred to polyvinylidene fluoride (PVDF) membranes (Millipore, IN, USA) using a mini trans-blot (Hercules, CA, USA). Membranes were then blocked with PBST (PBS with 0.05% Tween 20) containing 5% non-fat dry milk for 1 h and incubated at 4°C overnight with anti-PTEN or anti-phospho-AKT polyclonal antibody (Santa Cruz Biotechnology, CA, USA), anti-Bcl-xL polyclonal antibody (Cell Signaling, CA, USA) in fresh blocking buffer. Membranes were then washed with PBST and incubated with horseradish peroxidase-conjugated secondary antibody (Santa Cruz Biotechnology, USA) for 1 h. The blots were developed using an enhanced chemiluminescence (ECL) kit (Pierce, IL, USA). Protein levels were normalized against β-actin (Sigma-Aldrich, MO, USA).

### Anti-miRNA oligonucleotide transfection

HaCaT cells were transfected with 30 nM anti-miR^™^ miR-141 inhibitor or 30 nM of anti-miR^™^ miRNA inhibitor Negative control #1 (Control) (Ambion, TX, USA) using Lipofectamine 2000 (Invitrogen, USA) following the manufacturer's protocol. The cells were treated and analyzed 24 h after transfection.

### Statistical analysis

Data were shown as mean±SE, the SPSS software package (Version 10.0; SPSS Inc., Chicago, IL) was used for the statistical analyses. All data were analyzed statistically using the Student's *t*-test, with *P* values less than 0.05 considered statistically significant.

## RESULTS

### Effects of UVB irradiation on miR-141 expression

**Fig. 1:**
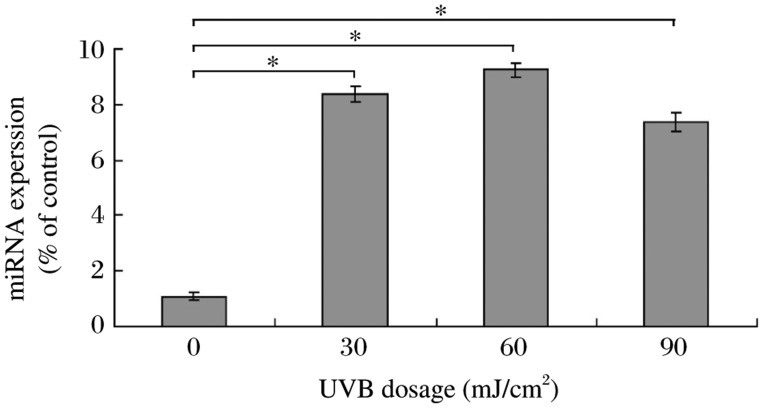
miR-141 expression in UVB-treated HaCaT cells. Total RNA was isolated from HaCaT cells treated with different doses of UVB. Equal amounts of total RNA from each sample were subjected to reverse transcription using primers designed specifically against miR141, followed by quantitative polymerase chain reaction (qPCR). The level of mature miR-141 was significantly increased in UVB-irradiated cells compared to untreated cells. The results show data from at least three independent experiments, *P < 0.05.

The mechanism by which UVB radiation inhibits PTEN expression in HaCaT cells is unknown. Previously, PTEN was shown to be a target of miR-141[15]. Therefore, we investigated miR-141 levels in UVB-treated cells. Our real-time RT-PCR results indicated that miR-141 expression was significantly upregulated at all UVB dosages examined (30, 60 and 90 mJ/cm^2^; ***Fig. 1***) compared to control-treated cells (0 mJ/cm^2^).

### UVB irradiation downregulates PTEN in HaCaT cells

**Fig. 2:**
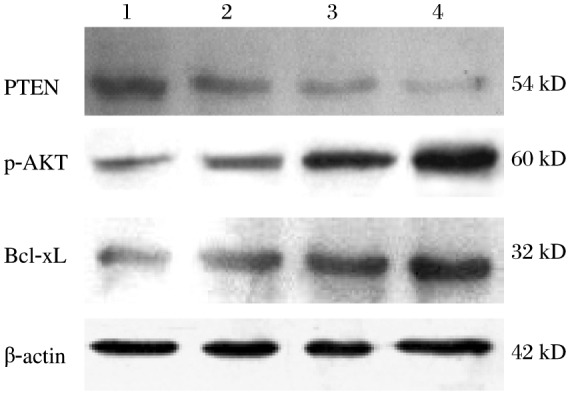
Effects of UVB treatment on PTEN protein expression. HaCaT cells were treated with different UVB concentrations and PTEN, P-AKT, and Bcl-xL levels were assayed by Western blotting. Equal amounts of total cellular protein were resolved by 10% SDS-PAGE, and β-actin was used as an internal control. Lane 1: untreated control group; Lane 2: 30 mJ/cm^2^ UVB-treated group; Lane 3: 60 mJ/cm^2^ UVB-treated group; Lane 4: 90 mJ/cm^2^ UVB-treated group.

As shown in ***Fig. 2***, there was significant dose-dependent decrease in the level of PTEN expression in UVB-treated HaCaT cells. Quantitative analysis indicated that 24 h after UVB exposure of 30, 60 or 90 mJ/cm^2^, PTEN expression was reduced to 76.43%, 31.45%, and 17.37% of control levels, respectively.

### UVB can induce apoptosis in HaCaT cells

**Fig. 3:**
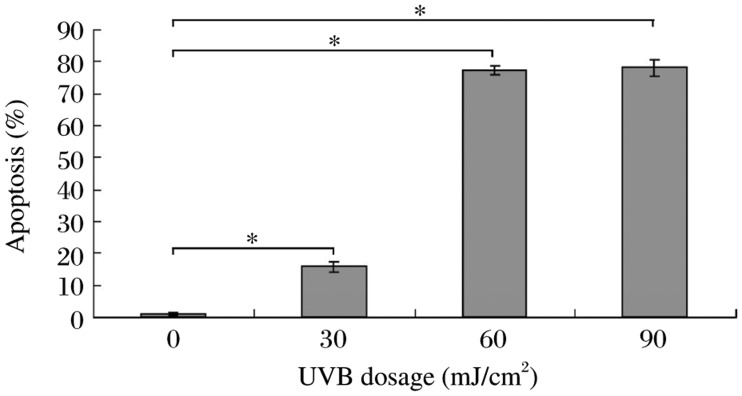
Effects of UVB treatment on apoptosis in HaCaT cells. Apoptosis rates after UVB treatment at various dosages. After 24 h of culture, annexin V/fluorescein isothiocyanate (FITC) staining was performed, and cells were analyzed by flow cytometry, *P < 0.05.

UVB had a dose-dependent cytotoxic effect on HaCaT cells, as revealed by our annexin V/PI staining assay results (***Fig. 3***). Treatment with 30-90 mJ/cm^2^ UVB induced significantly higher apoptosis rates (78.18% of cells were apoptotic at the 90 mJ/cm^2^ UVB dose) compared with controls 24 h after irradiation.

### Anti-miR-141 restored PTEN expression

**Fig. 4:**
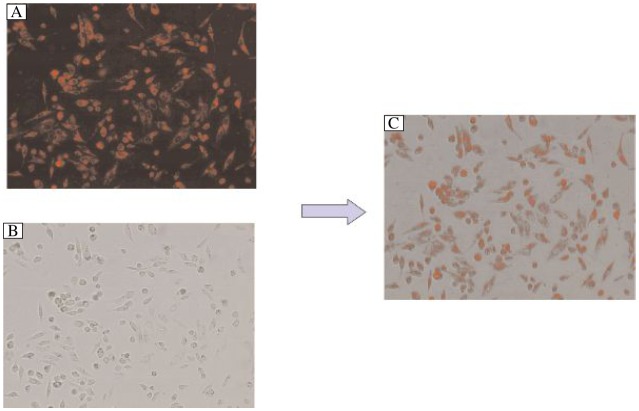
Anti-miRNA oligonucleotide transfection of HaCaT cells. The anti-miRTM miR-141 inhibitor was marked by TRITC (tetramethyl rhodamine isothiocyanate). Twenty-four h after transfection, we observed HaCaT cells via fluorescence microscopy (×200), over 90% of cells contained the anti-miRNA oligonucleotide. A: Image observed by fluorescence microscopy. B: Image observed under microscope. C: merging of A and B.

**Fig. 5:**
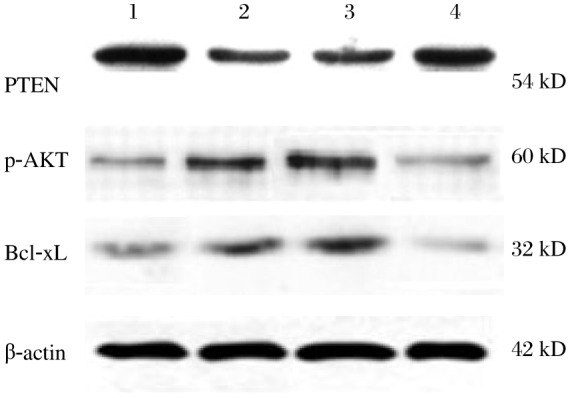
Inhibition of miR-141 restores PTEN levels. HaCaT cells were transfected with miR-141 inhibitors and treated with 30 mJ/cm^2^ UVB. PTEN, p-AKT and Bcl-xL expression were assessed by Western blotting 24 h after UVB irradiation. Equal amounts of total cellular protein were resolved by 10% SDS-PAGE, and β-actin was used as an internal control. Lane 1: untreated control group; Lane 2: UVB treated group; Lane 3: UVB+ anti-miR-141 negative control group; Lane 4: UVB+ anti-miR-141 group.

**Fig. 6:**
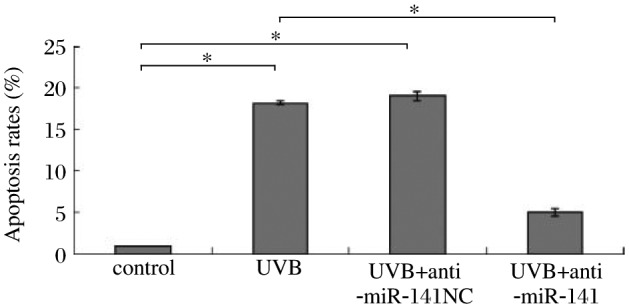
Inhibition of miR-141 decreases UVB-induced apoptosis. HaCaT cells were transfected with miR-141 inhibitors and treated with 30 mJ/cm^2^ UVB. After 24 h of culture, annexin V/fluorescein isothiocyanate (FITC) staining was performed, and cells were analyzed by flow cytometry, *P < 0.05.

To further confirm that miR-141 can affect PTEN protein levels in HaCaT cells, we transfected HaCaT cells with anti-miR-141 oligonucleotides (***Fig. 4***), then treated them with 30 mJ/cm^2^ UVB. PTEN levels were determined by Western blotting 24 h after treatment. The results suggest that transfection of UVB-irradiated HaCaT cells with anti-miR-141 efficiently abolished the inhibitory effect of miR-141 (***Fig. 5***). Moreover, the annexin V/PI staining assay revealed that anti-miR-141 decreased apoptosis in UVB-irradiated HaCaT cells (***Fig. 6***), suggesting that miR-141 is a likely regulator of PTEN expression. In other words, anti-miR-141 can partially restore the expression of PTEN and reverse the pro-apoptosis effect of UVB (***Fig. 5*** and ***Fig. 6***).

### Decreased PTEN expression induces AKT activation and Bcl-xL upregulation

Furthermore, in order to make clear the effect of PTEN in the apoptosis induced by UVB, we detected the phospho-AKT and Bcl-xL expression of each group, As shown in ***Fig. 2*** and ***Fig. 5***, the lever of phospho-AKT and Bcl-xL proteins were increased when the PTEN expression was down- regulated. These results indicated that PTEN-AKT-Bcl-xL could play an important part in the apoptosis of HaCaT cells and may be regulated by UVB and miR-141.

## DISCUSSION

Damage to skin cells from repeated UVB exposure can lead to cancer[18]–[20]. Cellular mechanisms exist to repair the DNA damage, or induce apoptosis to remove severely damaged cells[21],[22]. The molecular events involved in the induction of skin cancer are under active investigation, and until recently most studies have focused on protein-coding genes. However, several studies have now shown that UVB can regulate the expression of many miRNAs related to cell proliferation, differentiation, apoptosis and DNA damage response[6]–[8], indicating that miRNAs play an important role in UVB-mediated responses.

The PTEN gene, located at 10q23.3, encodes a central domain with homology to the catalytic region of protein tyrosine phosphatases[23]. This gene is an important regulator of protein phosphatases and 3′-phosphoinositol phosphatases[24]. PTEN dephosphorylates phosphatidylinositol-3,4,5-triphosphate (PIP3), the second messenger produced by phosphoinositide 3-kinase (PI3K), to negatively regulate the activity of the serine/threonine protein kinase, Akt[25]–[27]. PTEN is inactivated in some malignant tumors, resulting in Akt hyper-activation, thereby promoting cell proliferation, inhibition of apoptosis, and enhancing cell invasion and radioresistance[28]–[30]. In addition, UVB irradiation has been shown to increase PTEN and Akt phosphorylation in human dermal fibroblasts, which in turn leads to UVB-induced secretion of MMP-1 and -3, enzymes crucial for UV irradiation-induced photoaging in human skin[31]. These results indicate that PTEN is a target of UVB radiation and that it is involved in the pathology of photodamage. In this study, we also found that phospho-AKT and Bcl-xL expression was increased when the PTEN expression was downregulated, suggesting that the PTEN-AKT signal pathway could play a role in photodamage induced by UVB radiation in HaCaT cells.

In our previous study, we assessed the effect of UVB irradiation on miRNA expression in HaCaT cells and found that miR-141 was up-regulated in UVB-irradiated cells compared to controls. Using TargetScan and GO-analysis to predict miR-141 targets, we found that miR-141 may be involved in photocarcinogenesis, hypomethylation and apoptosis, suggesting that miR-141 is involved in the pathogenesis of photodamage. In the present study, we confirmed that miR-141 expression increased in a dose-dependent manner in UVB-treated cells. Furthermore, we found that upregulation of miR-141 expression after UVB radiation is inversely correlated with PTEN expression in HaCaT cells, which is accompanied by increased apoptosis.

Recently, UVB has been shown to down-regulate PTEN in apoptotic cells through degradation by active caspases, and by suppressing PTEN transcription in surviving cells[17]. The seed sequence of miR-141 was shown to match the 3′UTR of PTEN, and PTEN-3′UTR luciferase reporter assays confirmed PTEN as a direct target of miR-141[15]. To further investigate the mechanism underlying PTEN downregulation, we employed anti-miRs that efficiently and specifically silence endogenous miRNAs. Anti-miR-141 is a specific inhibitor of miR-141. Our results indicated that anti-miR-141 increased PTEN expression and decreased apoptosis in UVB-treated cells. Since inhibition of miR-141 has also been shown to reduce the apoptosis rate in nasopharyngeal carcinoma cells[15], we suggest that decreased apoptosis rate in UVB-treated cells can be attributed to the downregulation of miR-141. However, the decreased apoptosis rate may also contribute to increased PTEN expression following miR-141 inhibition, as PTEN is cleaved by active caspase in apoptotic cells[17].

Because gene expression is regulated via a complex cellular network, it is likely that many factors other than miR-141 are involved in the regulation of PTEN expression. However, we conclude that UVB suppresses the expression of PTEN by upregulating miR-141 in HaCaT cells. Furthermore, the results of this study indicate that miR-141 could serve as a potential gene therapy target for UVB-induced photodamage.
